# Screening for *Rickettsia*, *Coxiella* and *Borrelia* Species in Ticks from Queensland, Australia

**DOI:** 10.3390/pathogens9121016

**Published:** 2020-12-02

**Authors:** Hazizul Hussain-Yusuf, John Stenos, Gemma Vincent, Amy Shima, Sandra Abell, Noel D. Preece, Mythili Tadepalli, Sze Fui Hii, Naomi Bowie, Kate Mitram, Stephen Graves

**Affiliations:** 1 Australian Rickettsial Reference Laboratory, Geelong University Hospital, Geelong 3216, Victoria, Australia; hazizul.hussain-yusef@barwonhealth.org.au (H.H.-Y.); gemma.vincent@barwonhealth.org.au (G.V.); mythili.tadepalli@barwonhealth.org.au (M.T.); sze.hii@unimelb.edu.au (S.F.H.); graves.rickettsia@gmail.com (S.G.); 2 Centre for Tropical Environmental and Sustainability Science, James Cook University, Townsville 4611, Queensland, Australia; amyshima3@gmail.com (A.S.); noel.preece@jcu.edu.au (N.D.P.); 3 Centre for Tropical Biodiversity and Climate Change, James Cook University, Townsville 4611, Queensland, Australia; sandra.abell@jcu.edu.au (S.A.); naomi.bowie@my.jcu.edu.au (N.B.); kate.mitram@my.jcu.edu.au (K.M.); 4 Research Institute for Environment and Livelihoods, Charles Darwin University, Darwin 0815, Northern Territory, Australia; 5 Department of Microbiology and Infectious Diseases, Nepean Hospital, NSW Health Pathology, Penrith 2747, New South Wales, Australia

**Keywords:** *Ixodes*, *Amblyomma*, *Haemaphysalis*, *Rhipicephalus*, molecular detection, PCR, Lyme disease

## Abstract

Tick bites in Australia are linked to the transmission of a variety of infectious diseases in humans, livestock and wildlife. Despite this recognition, little is currently known about the variety of potential pathogens that are carried and transmitted by Australian ticks. In this study, we attempted to expand knowledge of Australian tick-borne bacterial pathogens by analyzing various tick species from the state of Queensland for potential human pathogens belonging to the *Rickettsia*, *Coxiella* and *Borrelia* genera. A total of 203 ticks, comprising of four genera and nine different tick species, were screened by specific qPCR assays. An overall *Rickettsia* qPCR positivity of 6.4% (13/203) was detected with rickettsial DNA found in four tick species (*Ixodes holocyclus*, *I. tasmani*, *Amblyomma* *triguttatum*, and *Haemaphysalis longicornis*). Amplification and analysis of several rickettsial genes from rickettsial qPCR positive samples identified sequences closely related to but genetically distinct from several previously described cultured and uncultured rickettsial species in the *Rickettsia* spotted fever subgroup. No ticks were positive for either *Coxiella* or *Borrelia* DNA. This work suggests that a further diversity of rickettsiae remain to be described in Australian ticks with the full importance of these bacteria to human and animal health yet to be elucidated.

## 1. Introduction

Ticks are arthropods with a complex life history that involves four stages, three which require a blood meal from a vertebrate animal host prior to metamorphosing into the next developmental stage [1,2]. When ticks parasitize humans, blood feeding creates the potential for tick microorganisms (e.g., bacteria, viruses, protozoa) to be transmitted to the human host. As a result of this activity, ticks are globally recognized as important vectors of infectious diseases in humans and animals.

In Australia, tick bites have been associated with a range of important infectious diseases, including ‘Q fever’ (*Coxiella burnetii)*, Queensland tick typhus (*Rickettsia australis)*, Flinders Island spotted fever (*Rickettsia honei*) and Australian spotted fever (*Rickettsia honei* subsp. *marmionii*) [1,3]. In Australia, 70 species of ticks have been identified, of which 17 are known to bite humans [4,5]. Of these, the three species that are considered the most important for transmission of bacterial infections are: (i) the paralysis tick (*Ixodes holocyclus*), the known vector for Queensland tick typhus and Q fever; (ii) the ornate kangaroo tick (*Amblyomma triguttatum*), another vector of Q fever and; (iii) the southern reptile tick (*Bothriocroton hydrosauri*), the vector of Flinders Island spotted fever [1].

In addition to these well recognized tick-borne infectious diseases, there are claims by medical practitioners and patient groups in Australia that other tick-borne diseases such as Lyme Disease, caused by *Borrelia burgdorferi sensu lato*, are widespread and cause significant illness in the community [6]. Previous analysis of *I. holocyclus* ticks from the eastern central coast of Australia (New South Wales), detected a number of potential human bacterial pathogens in this tick species, but not the causative agents of Lyme Disease (*Borrelia spp*) [7]. A recent bacterial community analysis of an expanded range of Australian wildlife ticks also failed to find evidence for *B. burgdorferi* and instead identified only novel *Borrelia* and other bacterial sequences [8].

Public health responses to tick-borne diseases rely on up-to-date knowledge of the types of ticks present in a given area, the life cycle stages that may be associated with pathogen transmission and the diversity of potential human and animal pathogens found in the ticks themselves [9]. Since ticks also move between the wildlife/domesticated animal interface, similar information is also potentially important for native species conservation and protection of livestock production systems [10,11].

In the current investigation, we moved to fill gaps in knowledge about the identity and prevalence of tick-borne bacteria and potential pathogens of humans and animals in Australia by conducting specific screening for the presence of *Rickettsia*, *Coxiella*, and *Borrelia* in Australian ticks collected from north-eastern Australia (Queensland).

## 2. Results

### 2.1. Tick Identification

Ticks (*n* = 203) from a variety of species were sampled as a part of this investigation (Table 1). Following the process of identification, the majority of ticks were found to belong to the genus *Ixodes* (*n* = 157), including *Ixodes hirsti* (*n* = 1), *I. holocyclus* (*n* = 134), *I. tasmani* (*n* = 17). Similar numbers of *Rhipicephalus* (*n* = 21) and *Haemaphysalis* (*n* = 20) ticks were identified. Species from the genus *Rhipicephalus*, were limited to *R. sanguineus*. *Haemaphysalis* species ticks were comprised of *Haemaphysalis bancrofti* (*n* = 10), *Haemaphysalis humerosa* (*n* = 2), *Haemaphysalis longicornis* (*n* = 3), and *Haemaphysalis novoguineae* (*n* = 1). *Amblyomma triguttatum* ticks were also found (*n* = 2). A further five *Ixodes* ticks, four *Haemaphysalis* and three *Amblyomma* ticks could not be identified at the species level.

Ticks were removed from a variety of hosts, including humans, domesticated animals and wildlife (Table 1). From placental hosts, ticks were mainly available from humans and dogs with a small number of individual ticks collected from other sources (e.g., cats, melomys). Ticks from humans were mainly *I. holocyclus*. Dog ticks were largely divided between *I. holocyclus* and *R. sanguineus*, the latter only retrieved from this host. In terms of marsupial hosts, nearly half of the ticks sampled from marsupials (*n* = 89) were removed from tree kangaroos (*n* = 39) with even numbers of ticks (*n* = 18) each taken from bandicoots and bettongs. Notably, bettongs also carried the highest diversity of tick species detected. Unlike the other marsupial hosts where *I. holocyclus* were the most common ticks, *H. bancrofti* was the most common tick retrieved from bettongs.

### 2.2. PCR Detection of Bacterial Species in Ticks

qPCR assays specific for *Rickettsia*, *Coxiella* and *Borrelia* were used to detect the presence of bacterial DNA in the ticks sampled in this study from Queensland, Australia.

*Rickettsia* spp. qPCR revealed an overall positivity of 6.4% (13/203) for rickettsial DNA. The distribution of rickettsial PCR positivity across different tick species and the hosts they were retrieved from is presented in Table 2. PCR positivity was slightly higher in ticks retrieved from marsupial hosts (9.0%; 8/89) than those from placental mammals (5.7%; 5/88). Among the tick species screened, PCR positivity was highest for *Amblyomma* spp. ticks (80%; 4/5) although the total number of ticks screened was small. Rickettsial DNA was most commonly found in ticks from the genus *Ixodes* with an overall prevalence of 5.1% (8/157). Within this tick genus, *I. tasmani* had the highest prevalence (29.4%; 5/17) followed by *I. holocyclus* (2.2%; 3/134). All *Rhipicephalus* ticks were negative by the rickettsial PCR (0/21).

In ticks from marsupial hosts, rickettsial PCR positivity was limited to bandicoots (16.7%; 3/18), bettongs (22.2%; 4/18) and quolls (20.0%; 1/5). For the ticks from domesticated animals, a small number of ticks from dogs (4.7%; 2/43) and cats (25.0%; 1/4) were found to be *Rickettsia* spp. qPCR positive. Five percent of ticks (2/40) retrieved from humans were PCR positive for rickettsial DNA. All ticks were PCR negative by *Coxiella* and *Borrelia* specific qPCR assays

### 2.3. Sequence Based Rickettsia spp. Identification in PCR Positive Tick Samples

Conventional PCR amplification and sequencing of rickettsial genes was used to determine the species-level identity of all *Rickettsia* qPCR positive samples (*n* = 13). Results from this analysis are presented in Table 3.

Initial attempts focused on PCR amplification of the *Rickettsia gltA* gene led to successful amplification of 7/13 partial *Rickettsia gltA* sequences. Following manual curation, BLAST analysis was used to determine the likely sequence identity. Four sequences from *I. tasmani* (bettong, melomys and cat hosts) and *I. holocyclus* ticks (canine host) were found to share 100% sequence identity to the partial *gltA* gene sequence for *Candidatus* Rickettsia antechini (Genbank Accession DQ372954.1) first reported in ectoparasites removed from the small Australian marsupial, the yellow-footed antechinus (*Antechinus flavipes*). The *gltA* sequences amplified from *Amblyomma* spp. ticks removed from bettongs and a human, were found to share between 99.7–100% sequence similarity to partial *gltA* sequences from *Rickettsia gravesii* (DQ269435.1), previously detected in *A. triguttatum* ticks [12].

Further attempts to determine the identity of these *Rickettsia* positive samples were made by PCR amplification and sequencing of the partial *Rickettsia 17kDa*, *ompB* and s*ca4* genes. These attempts were not as successful as initial *gltA* PCRs with only five partial *17kDa* gene, one partial *ompB* and two partial *sca4* genes amplified and sequenced from the tick DNA extracts (Table 3). BLAST analysis revealed similarity to deposited sequences previously amplified from a range of putatively and formally classified rickettsiae detected in ticks, including (i) *17kDa* sequences (*n* = 4) from *Rickettsia raoultii* (99.5–99.8% similarity; MH932036.1) (ii) *ompB* sequences from Ca. Rickettsia antechini (99.8%; DQ372956.1); (iii) *sca4* sequences from *Candidatus* Rickettsia tasmanensis (99.4%; GQ223394.1) and *R. gravesii* (99.2%; DQ269439.1).

## 3. Discussion

This study provides molecular evidence for the presence of a variety of genetically distinct *Rickettsia* spp. from three different genera of ticks from north-eastern Australia. The *Rickettsia* positive ticks were removed from humans, domesticated animals and several species of wildlife, illustrating the potential for ticks to mediate the transmission of different tick-borne microorganisms across different host groups in this region.

The overall prevalence of *Rickettsia* spp. DNA detected in ticks from this Queensland study was a surprisingly low 6%. The study of rickettsiae in Australian ticks goes back to early observations of human infections following tick bites in this region of Australia. The infection was named Queensland Tick Typhus and the responsible tick species were shown to be *I. holocyclus* and *I. tasmani* [13,14]. Two recent molecular studies performed concurrently to our own work found an overall rickettsial PCR prevalence of 13% in ticks removed from domesticated animals (horses, in particular) and wildlife from Central Queensland [15] and 15.4% from *I. holocyclus* ticks removed from the environment in North-Eastern New South Wales [16], rates slightly higher than that described in our study. In the former [15], *Rickettsia* spp. were also found in several genera of ticks, including *Haemaphysalis*, *Rhipicephalus*, *Ixodes*, and *Amblyomma* amongst others. Excepting *Rhipicephalus*, we also detected *Rickettsia* DNA in ticks from these genera. The prevalence rates documented in these studies of rickettsial prevalence in ticks removed from warm-blooded animals stands in contrast to recent surveys of high rickettsial positivity (92–100%) in ticks removed from reptiles [17,18]. This difference incidence raises questions over the exact relationship that the detected rickettsias have with their tick hosts. An incidence as high as has been reported in reptile ticks could suggest that the detected species (e.g., closely related to existing species in the Rickettsia spotted fever group [17,18] may be in an endosymbiotic relationship with their reptile tick host. The lower positivity in hard ticks removed from warm-blooded animals, however, might suggest that these Rickettsia are simply commensals. While the latter discussion is clearly speculative, the data in this study nevertheless suggests that, while uncommon, *Rickettsia* infect a diverse range of hard tick taxa that can parasitise both domesticated animals and wildlife in north-eastern Australia.

The previously published Queensland [15] and New South Wales [16] studies also used sequence analysis of the same gene targets to determine the potential identity of *Rickettsia* species. In the Queensland study, several rickettsia sequences were amplified that shared homology to sequences from *R. gravesii* but also *Rickettsia argasii*, *Rickettsia japonica*, and uncultured rickettsiae [15]. In the New South Wales study, however, the only rickettsial sequences amplified belonged to *Rickettsia australis*, the bacterium causing Queensland tick typhus [16]. In our study, we found sequences that shared homology to deposited sequences from a variety of formally classified rickettsial agents, including *R. gravesii* and *R. raoultii*, as well as previously detected but unclassified *Rickettsia* spp., including Ca. *R. antechini*, and Ca. *R. tasmanensis*. *R. gravesii* was first reported in high prevalence in *A. triguttatum* ticks in Western Australia [12]. *R. raoultii* is an emerging infectious disease agent in Europe and Asia associated with tick-borne lymphadenopathy [19,20]. It has been found at a high prevalence in *Dermacentor* ticks [21]. Ca. R. antechini sequences were deposited from an unpublished study of novel *Rickettsia* spp. in the ground-dwelling marsupial, *Antechinus flavipes*, in Western Australia while Ca. *R. tasmanensis* sequences were first reported in ticks from Tasmania [22]. It is important to acknowledge that our sequence-based identifications are based on BLAST homology to partial gene sequences deposited in GenBank. It is hence possible that these sequences may not represent the detection of the aforementioned rickettsial species but rather novel Australian rickettsiae. The results of this work nevertheless indicate that there is diversity of genetically distinct *Rickettsia* spp. that remain to be fully explored in Australian ticks.

No PCR positivity for *Borrelia* species was found in the ticks screened in this study. The presence or absence of Lyme disease, caused by *Borrelia* spp., in Australia is a controversial subject. Previous investigations utilizing a range of methods including culture, PCR and/or next generation sequencing have failed to provide convincing evidence for Lyme Disease-causing *Borrelia* spp. in Australian ticks [7,23,24,25,26]. DNA from other *Borrelia* species has been detected, including (i) *Borrelia queenslandica*, associated with the kangaroo soft tick *Ornithodoros gurneyi*, and may be present in certain native rats such as *Rattus villosissimus* [27]; (ii) uncultured *Borrelia* spp. in *I. holocyclus* and *Bothriocroton concolor* ticks from monotremes that are genetically distinct from *B. burgdorferi* (senus lato) [7,28]. This study adds to the growing evidence that Lyme disease causing *Borrelia* spp. are not found in Australian tick species.

*C. burnetii*, the bacterium causing Q Fever in humans and coxiellosis in animals, was first recognized in a tick in the USA [29]. *C. burnetii* has subsequently been detected in various Australian tick species, including studies of ticks from Queensland, such as *I. holocyclus* [30,31], *A. triguttatum* [30,32] and *H. humerosa*, the latter retrieved from bandicoots (*Isoodon torosus*) on Moreton Island off the east coast of Queensland [33]. Coxiella DNA related to *C. burnetii* has also been detected in the tick, *Bothriocroton auruginans*, from wombats [34]. In this study, none of the ticks tested was positive for *C. burnetii.* This was somewhat surprising given that previous PCR studies of ticks from northern Queensland revealed ca. 30% PCR positivity for *C. burnetii* in *A. triguttatum* and *I. holocyclus* ticks removed from similar wildlife species as those sampled in this study [30]. *C. burnetii* DNA was also found in *I. holocyclus* removed from domestic and native animals, albeit at a lower prevalence of 6% [31]. Quantification of *C. burnetii* genome equivalents in ticks suggests that the infectious load is only very low [30]. As such, it is possible that very low levels of *C. burnetii* present may have gone undetected in ticks screened by our molecular approach.

The most obvious limitation of our study was the ad-hoc nature of sample collection whereby the tick collection screened was assembled over a period of four years (2013–2016) from a variety of geographic locations and host sources in Queensland. Sampling was not systematic or exhaustive meaning that sample sizes for certain tick species were very small and the variety of potential hosts was also under-sampled. As a result, an accurate estimate of the prevalence of *Rickettsia* or, indeed, *Coxiella* and *Borrelia* infections in ticks from Queensland remains to be elucidated with the results of the current study providing a snapshot to guide more thorough systematic investigations.

## 4. Materials and Methods

### 4.1. Tick Collection and Identification

Ticks collected from human, domestic animals and wildlife were provided to the Australian Rickettsial Reference Laboratory (ARRL) on an ad-hoc basis by collaborating colleagues, other laboratories, medical practitioners and patients from the state of Queensland in north-eastern Australia. The majority of ticks originated from the Tablelands region of Far North Queensland (17.2661° S, 145.4859° E) with a smaller selection of samples from other locales in southern and western Queensland. Ticks were either preserved in 70% ethanol or placed alive or dead in small plastic vials and posted to the ARRL. Ticks were then identified morphologically according to previously published tick identification keys [4,35].

### 4.2. DNA Extraction and PCR Detection of Rickettsia, Borellia and Coxiella spp.

After identification, each tick was individually cleaned with phosphate-buffered saline (PBS) and homogenized in a 1.5 mL microcentrifuge tube containing 300 μL of PBS using a micropestle. Total genomic DNA was extracted from 100 μL of the tick homogenate using a commercial kit (Real Genomics DNA extraction kit, Real Biotechnology Corporation, ChungHo, Taiwan), according to the manufacturer’s tissue protocol. DNA was eluted in 100 μL of elution buffer.

*Rickettsia* spp. detection was performed by real-time qPCR assays targeting the citrate synthase (*gltA*) gene of the spotted fever (SFG) and typhus (TG) groups [36]. *C. burnetii* was detected using primers targeting the *C. burnetii* outer membrane protein *com1* gene [37] and *htpAB* heat shock operon from *C. burnetii* [38]. All oligonucleotides were supplied by Biosearch Technologies (Novato, CA, USA). Amplification of all three genes was performed using the following cycling protocol; one step at 50 °C for 2 min, one step at 95 °C for 2 min and 40 cycles at 95 °C for 10 s and 60 °C for 20 s. DNA from *R. australis* str. Phillips was used as a positive control for *Rickettsia* spp. qPCR assays. The *C. burnetti* str. Henzerling was used as the positive control for *C. burnetii* qPCR assays.

A SYBR Green real-time qPCR assay was used to detect the Lyme disease causing species of *Borrelia (B. burgdorferi* sensu stricto, *Burgdorferi afzeli* and *Burgdorferi garinii*), targeting the *recA* gene [39]. Control Borrelia DNA from these species was obtained from Dr Volker Fingerle, the head of German National Reference Centre for Borrelia, Erlangen, Germany. After amplification, melt curve analysis allowed differentiation of the species.

### 4.3. Confirmation of PCR Positive Results and Species Identification by DNA Sequencing

Samples that were positive in the *gltA* assay were subjected to further testing in triplicate to confirm positive results and to possibly identify the *Rickettsia* species. A larger fragment (382 bp) of the *gltA* gene [40], plus a fragment (450 bp) of the *17kDa* (outer membrane antigen), *sca4* (cell surface antigen) [41] and *ompB* (outer membrane protein-B) genes [42] were amplified by conventional PCR. Amplified DNA was purified using the Wizard SV gel and PCR clean-up system (Promega, Madison, WI, USA), and sent to Macrogen (Seoul, Korea) for sequencing. The sequence data was analysed using 4peaks (Version 1.8; http://nucleobytes.com) and the NCBI Blast software (http://blast.ncbi.nlm.nih.gov/Blast.cgi). Amplified sequences from this study are available on GenBank (MT277097–MT277110).

## 5. Conclusions

This study surveyed four genera of ticks (nine species) for the presence of *Rickettsia*, *Coxiella* and *Borrelia* species by PCR. Five tick species contained rickettsial DNA, *I. holocyclus*, *I. tasmani*, *Amblyomma sp.*, *H. longicornis* and *A. triguttatum*) with an overall and relatively low prevalence of 6%, while *Coxiella* spp. or *Borrelia* spp. DNA were not detected at all. Sequence-based analysis of the amplified rickettsial sequences suggest that the *Rickettsia* spp. detected are closely related but genetically distinct from several other rickettsial species previously described in Australian ticks and elsewhere. Further work on wildlife as reservoirs and the effects of these pathogenic organisms on humans, livestock and wildlife will become more important as the human-wildlife interface becomes ever more strained [10,11].

## Figures and Tables

**Table 1:** Tick species and their vertebrate hosts sampled in this study.

Host	Total Ticks	Tick (Genus/Species)						
		*Amblyomma* spp.	*Haemaphysalis* spp. *	*Haemaphysalis bancrofti*	*Ixodes* spp. **	*Ixodes holocyclus*	*Ixodes tasmani*	*Rhipicephalus sanguineus*
**Mammal** **Marsupial (*n* = 89)**								
Northern brown bandicoot (*Isoodon macrourus*)	18	0	3	2	0	9	4	0
Northern bettong *(Bettongia tropica)*	18	4	3	6	1	2	2	0
Possum (*Trichosurus vulpecula* and *T. v. johnstoni*)	5	0	0	0	1	4	0	0
Northern quoll (*Dasyurus hallucatus*)	5	0	0	0	0	2	3	0
Tree kangaroo (*Dendrolagus lumhotzii*)	39	0	2	0	0	32	5	0
Wallaby (*Macropus* sp)	4	0	0	2	1	1	0	0
**Placental (*n* = 88)**								
Cat (*Felis catus*)	4	0	0	0	0	3	1	0
Dog (*Canis lupis*)	43	0	1	0	1	20	0	21
Human (*Homo sapiens*)	40	1	1	0	1	37	0	0
Grassland melomys (*Melomys burtoni*)	1	0	0	0	0	0	1	0
Host-free	16	0	0	0	0	15	1	0
Host-unknown	10	0	0	0	1	9	0	0
**Total**	**203**	**5**	**10**	**10**	**6**	**134**	**17**	**21**

* Includes H. humerosa, H. longicornis, and H. novoguineae, but excluding H. bancrofti. ** Includes I. hirsti and Ixodes sp., excluding I. holocyclus and I. tasmani.

**Table 2:** Summary of *Rickettsia* spp. PCR screening results of ticks in this study.

	Ticks (*n*)	*Rickettsia* PCR Positivity *n* (%)
**Tick Species**		
*Amblyomma* (Total)	**5**	**4 (80.0)**
*Amblyomma triguttatum*	2	2 (100.0)
*Amblyomma* sp.	3	2 (66.7)
*Haemaphysalis* (Total)	**20**	**1 (5.0)**
*Haemaphysalis bancrofti*	10	0 (0.0)
*Haemaphysalis humerosa*	2	0 (0.0)
*Haemaphysalis longicornis*	3	1 (33.3)
*Haemaphysalis novoguineae*	1	0 (0.0)
*Haemaphysalis* sp.	4	0 (0.0)
*Ixodes* (Total)	**157**	**8 (5.1)**
*Ixodes hirsti*	1	0 (0.0)
*Ixodes holocyclus*	134	3 (2.2)
*Ixodes tasmani*	17	5 (29.4)
*Ixodes* sp.	5	0 (0.0
*Rhipicephalus* (Total)	**21**	**0 (0.0)**
*Rhipicephalus sanguineus*	21	0 (0.0)
**Host Species**		
Marsupial (*n* = 89)	**89**	**8 (9.0)**
Northern brown bandicoot (*Isoodon macrourus*)	18	3 (16.7)
Northern bettong *(Bettongia tropica)*	18	5 (22.2)
Possum (*Trichosurus vulpecula* and *T. v. johnstoni*)	5	0 (0.0)
Northern quoll (*Dasyurus hallucatus*)	5	1 (20.0)
Tree kangaroo (*Dendrolagus lumhotzii*)	39	0 (0.0)
Wallaby (*Macropus* sp.)	4	0 (0.0)
Placental (*n* = 88)	**88**	**5 (5.7)**
Cat (*Felis catus*)	4	1 (25.0)
Dog (*Canis lupus*)	43	3 (4.7)
Human (*Homo sapiens*)	40	2 (5.0)
Grassland melomys (*Melomys burtoni*)	1	0 (0.0)
Host-free	16	0 (0.0)
Host-unknown	10	0 (0.0)

**Table 3:** BLAST analysis of partial *Rickettsia gltA*, *17kD*, *ompB*, and s*ca4* gene sequences amplified from *Rickettsia* PCR positive samples (*n* = 13).

Sample ID	Tick Species	Host Species	BLAST Result *			
			*gltA*	*17kDa*	*ompB*	*Sca4*
2016-111	*I. tasmani*	Bandicoot	100% Ca. *R. antechini*	99.7% *R. raoultii*	NA ^	NA
2013.4-157	*I. tasmani*	Melomys	100% Ca. *R. antechini*	NA	NA	NA
2013.4-54-3	*I. holocyclus*	Dog	100% Ca. *R. antechini*	NA	99.8% Ca. *R. antechini*	NA
2013.4-140	*I. tasmani*	Cat	100% Ca. R. antechini	NA	NA	99.4% Ca. *R. tasmanensis*
2015-74	*Amblyomma* sp.	Human	100% *R. gravesii*	99.5% *R. raoultii*	NA	NA
2015-104	*Amblyomma* sp.	Bettong	99.7% *R. gravesii*	99.8% *R. raoultii*	NA	99.2% *R. gravesii*
2016-116	*A. triguttatum*	Bettong	99.7% *R. gravesii*	99.8% R. *raoultii*	NA	NA

* Highest percent identity BLAST hits are listed. ^ NA = no amplification.
